# No reduced patellar loading with latest-generation cruciate-retaining total knee arthroplasty—a comparative study of Attune and Press-Fit Condylar®

**DOI:** 10.1007/s00264-020-04717-4

**Published:** 2020-07-17

**Authors:** Filippo-Franco Schiapparelli, Puja Ahmadi, Felix Amsler, Michael T. Hirschmann

**Affiliations:** 1 Department of Orthopaedic Surgery and Traumatology, Kantonsspital Baselland (Bruderholz, Liestal, Laufen), Bruderholz, CH-4101 Basel, Switzerland; 2 Amsler Consulting, CH-4059 Basel, Switzerland; 3 University of Basel, Basel, Switzerland

**Keywords:** Anterior knee pain, Tuberosity-trochlear groove index, SPECT-CT

## Abstract

**Purpose:**

To investigate if the latest-generation cruciate-retaining total knee arthroplasty (CR-TKA) systems through more patella-friendly femoral trochlea reduce the patellar bone loading.

**Methods:**

Twenty patients who underwent Attune CR-TKA were matched with twenty-one patients who underwent Press-Fit Condylar® (PFC) CR-TKA. The patella was always preserved. The in vivo patellar loading was measured twice by two blinded observers and localised on an 8-quadrant grid on 1-year post-operatively SPECT/CT images. The position of the TKA components, patella height, thickness, tilt, and tibial tuberosity-trochlear groove index were measured in 3D CT. Knee function was assessed pre-operatively, at 12 and 24 months post-operatively with the knee society score (KSS). All data were compared between groups with the Mann-Whitney *U* test and within groups with Spearman’s correlation.

**Results:**

A significantly higher bone tracer uptake (BTU) was seen in the Attune group in the lateral non-articular patellar quadrants. No other significant differences of the BTU were seen. The post-operative KSS did not differ significantly. Spearman’s correlation showed no correlations between the significantly higher BTU of the lateral non-articular patellar quadrants and the position of the TKA and patellar measurements. All patellar measurements did not correlate with bone stress in SPECT/CT.

**Conclusion:**

No significant improvement in terms of in vivo patellar bone stress was seen with the latest-generation CR-TKA system. The increased bone stress at the non-articular lateral patellar quadrants of the Attune could be due to higher stabilising quadriceps forces.

## Introduction

Anterior knee pain (AKP) and poor range of motion are two of the most commonly experienced problems after total knee arthroplasty (TKA) [1, 2]. Patellofemoral problems resulting in patellar overloading are often responsible for AKP [2, 3]. AKP is due to numerous causes ranging from functional to mechanical ones such as suboptimal positioning of the femoral component, patella-unfriendly trochlear shape, tibial tuberosity-trochlear groove index (TT-TG), patellar height, tracking, and tilt [2, 4].

**Fig. 1:**
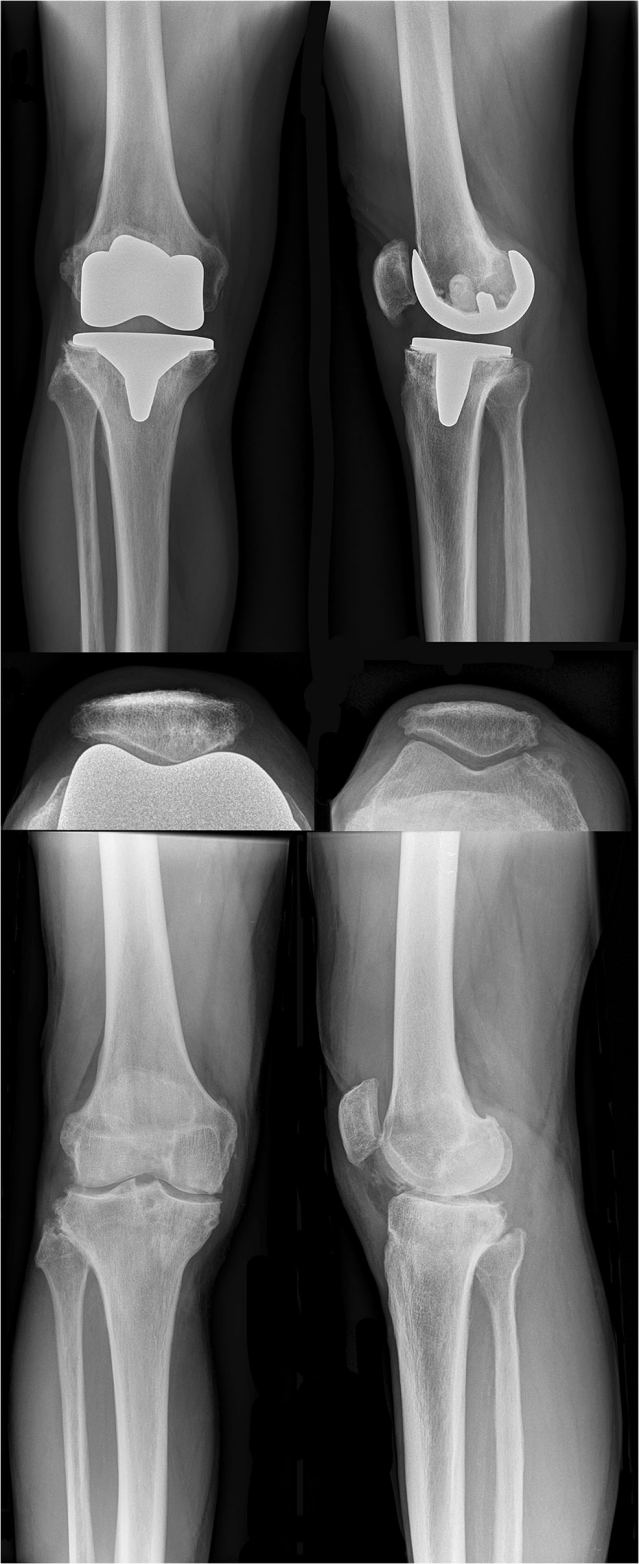
Pre- and post-operative radiographs of a patient who underwent Attune TKA. The standardised pre- and post-operative radiographs (ap, lateral, and patella tangential) of a patient who underwent Attune TKA are reported. Some differences in the femoral component with respect to the PFC can be appreciated, a shallower trochlear groove, a flatter trochlea, and the multi-radius shape of the femoral condyles. A good TKA component position and patella height are shown

**Fig. 2:**
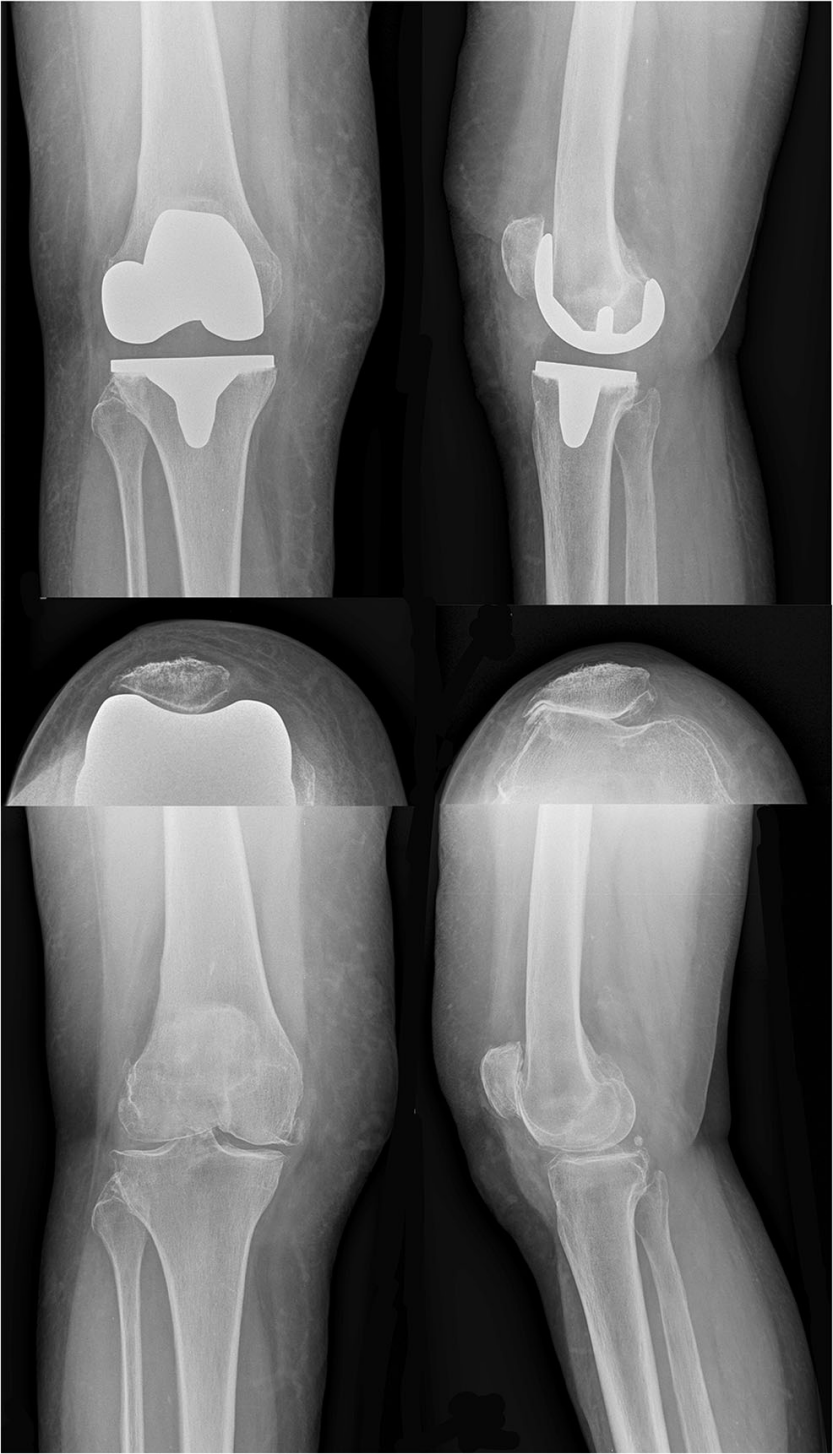
Pre- and post-operative radiographs of a patient who underwent PFC TKA. The standardised pre- and post-operative radiographs (ap, lateral, and patella tangential) of a patient who underwent PFC TKA are reported. A good TKA component position and patella height are shown

Aiming for less patients with AKP, orthopaedic surgeons work on an improved understanding of patellofemoral kinematics, a more optimal TKA positioning and more patella-friendly prosthetic components. The ATTUNE ® Primary Total Knee System (Attune TKA; DePuy Orthopaedics Inc., Warsaw, IN, USA) (Fig. 1), the latest-generation TKA and successor of the Press-Fit Condylar® Total Knee System (PFC TKA; DePuy Orthopaedics Inc., Warsaw, IN, USA), is such an example (Fig. 2). Through a 3-mm shallower trochlear groove, different trochlear angles along the whole trochlea, and a multi-radius shape of the femoral condyles, the manufacturer aimed to improve the patellar tracking as well as diminishing patellar bone stress [5, 6].

The question how the distribution and intensity of the patellar in vivo bone stress is changed is still open. Hybrid single-photon emission computerised tomography (SPECT) combined with CT (SPECT-CT) allows a direct window into bone metabolism, and significant correlation between increased bone tracer uptake (BTU) and bone stress has been reported. SPECT-CT is therefore increasingly used in the orthopaedic field. Several authors investigated painful native and operated knee joints with SPECT-CT, and typical distribution patterns of increased BTU have been identified for the most common knee pathology [4, 7–10].

The primary purpose of the present study was therefore, to compare the in vivo patellar bone stress between two different cruciate-retaining TKA (CR-TKA) systems without patellar resurfacing. The secondary purpose was to compare the pre-operative and post-operative knee function of the same patients at one and two years of follow-up.

The hypothesis of the study was to find less bone loading on the patella and similar or better post-operative knee function in the patients who underwent Attune CR-TKA.

## Material and methods

Twenty patients who underwent cruciate-retaining ATTUNE ® Primary Total Knee System (group A, male:female = 12:8, mean age ± standard deviation = 64.8 ± 9.6, right:left = 14:6) were matched with twenty-one patients who underwent cruciate-retaining Press-Fit Condylar® Total Knee System (group PFC, male:female = 12:9, mean age ± standard deviation = 69.7 ± 7.2, right:left = 14:7). The two groups showed no significant differences with respect to age, gender, and side (Table 1). The indication for CR-TKA was symptomatic primary bicompartmental osteoarthritis. All surgeries were performed through a standard medial parapatellar approach without patellar resurfacing from one surgical team. The choice not to resurface the patella was done intraoperatively based on a good shape of the patellar cartilage.

**Table 1:** In this table the two groups of patients are compared and tested for differences with regard to the demographics, to the patellar bone load (rmBTU) measured on SPECT images, to the pre- and post-operative functional assessment (KSS), and to the three-dimensional position of TKA components and patellar parameters measured on three-dimensional reconstructed CT images and standardised radiographs

			Group PFC mean ± SD	Group A mean ± SD	ICC	*p*
Demographics		Number of patients	21 (51.2%)	20 (48.8%)		
		Age (mean ± SD)	69.7 ± 7.2	64.8 ± 9.6		0.07
		Gender (*N*, %)	F: 9 (42.9%) M: 12 (57.1%)	F: 8 (40%) M: 12 (60%)		0.85
		Side (*N*, %)	Right: 14 (66.7%) Left: 7 (33.3%)	Right: 14 (70%) Left: 6 (30%)		0.81
		Time surgery-SPECT/CT (years)	1.18 ± 0.32	1.03 ± 0.09		0.16
SPECT-CT rmBTU	Articular quadrants	Medial superior (ams)	2.9 ± 1.72	2.66 ± 1.31	0.85	0.98
		Medial inferior (ami)	2.14 ± 1.95	2.07 ± 1.15	0.75	0.37
		Lateral superior (als)	2.34 ± 1.09	2.9 ± 1.3	0.88	0.12
		Lateral inferior (ali)	1.62 ± 0.99	1.92 ± 0.87	0.89	0.17
	Non-articular quadrants	Medial superior (nams)	2.02 ± 1.59	2.08 ± 1.26	0.88	0.37
		Medial inferior (nami)	1.18 ± 1.66	1.23 ± 0.71	0.84	0.06
		Lateral superior (nals)	1.55 ± 0.79	2.17 ± 1.09	0.88	0.03
		Lateral inferior (nali)	0.94 ± 0.79	1.21 ± 0.49	0.96	0.01
		Inferior quadrants (mean)	1.47 ± 1.29	1.61 ± 0.67		0.10
		Superior quadrants (mean)	2.2 ± 1.11	2.45 ± 1.11		0.42
KSS	Pre-operative	KSS knee	52.6 ± 13.1	60.9 ± 10.2		0.03
		KSS function	66.2 ± 18.6	80.5 ± 8.9		< 0.01
		KSS total	118.8 ± 23.9	141.4 ± 16.3		< 0.01
	Post-operative 12 months	KSS knee	91.7 ± 8.4	88.7 ± 9.4		0.30
		KSS function	95.6 ± 10.4	93 ± 10.8		0.46
		KSS total	187.2 ± 12.5	181.7 ± 16.5		0.25
	Post-operative 24 months	KSS knee	91.8 ± 8.6	87.6 ± 16		0.34
		KSS function	94.2 ± 8.4	88.6 ± 15.6		0.19
		KSS total	186.1 ± 14.9	176.2 ± 30.4		0.23
TKA component position	Femoral component	External (+) and internal (−) rotations	2.76° ± 3.03	2.85° ± 3.07		0.94
		Varus (+) and valgus (−)	0° ± 2.95	0.15° ± 1.69		0.72
		Flexion (+) and extension (−)	6.76° ± 3.74	9.8° ± 2.73		< 0.01
	Tibial component	External (+) and internal (−) rotations	− 6.33° ± 6.35	− 3.95° ± 4.76		0.23
		Varus (+) and valgus (−)	− 0.33° ± 2.18	2.15° ± 2.16		< 0.01
		Posterior (+) and anterior (−) slopes	4.52° ± 1.99	4.55° ± 2.65		0.77
	Patella	Tilt patella	1.47° ± 3.19	2.31° ± 2.36		0.30
		Thickness patella	26.75 ± 2.57	25.89 ± 3.43		0.24
		Modified Insall-Salvati index	1.58 ± 0.16	1.7 ± 0.12		0.01
		Caton-Deschamps index	0.7 ± 0.12	0.79 ± 0.14		0.03
		TT-TG	6.69 ± 4.94	7.17 ± 4.69		0.72

**Fig. 3:**
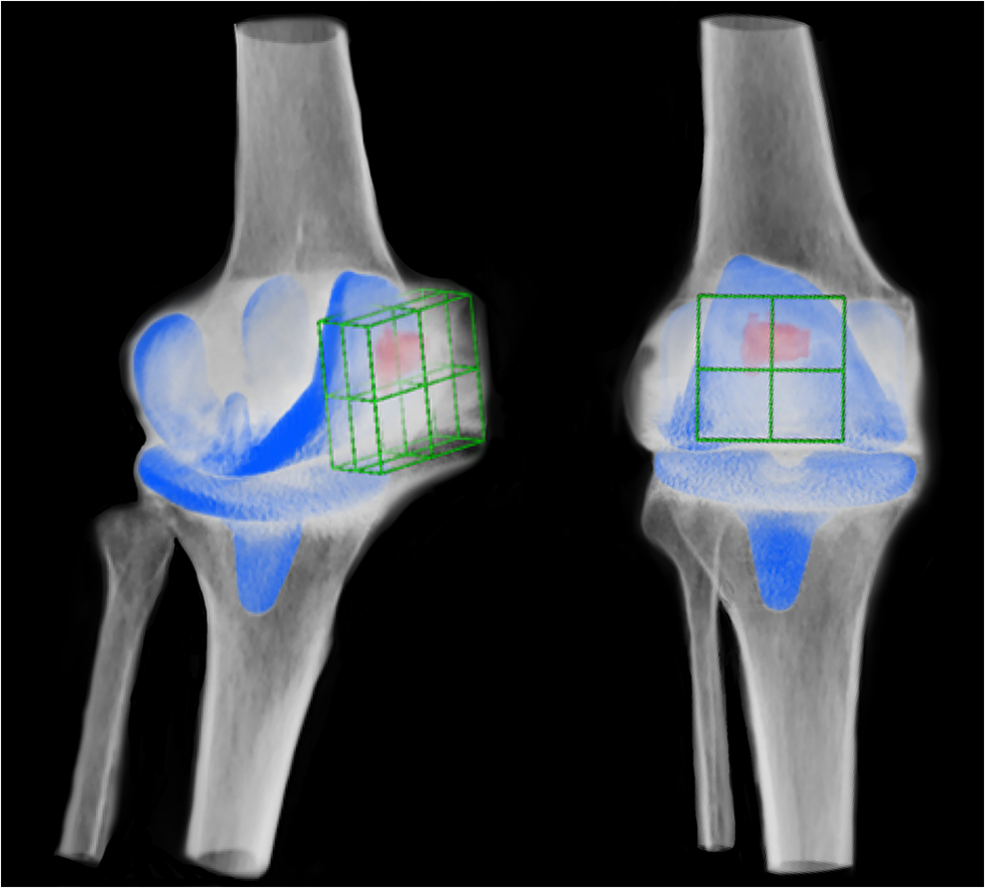
Three-dimensional reconstructed SPECT/CT images. An 8-quadrant patellar grid was used to measure and localise the in vivo bone stress using a customised software (OrthoExpert©, London, UK)

Every patient gave written informed consent to undergo serial clinical and radiological examinations with standard radiographs and SPECT/CT (time between TKA to SPECT/CT: group A 1.03 ± 0.09, group PFC 1.18 ± 0.32, *p*: 0.16) following a standardised protocol (Table 1, Figs. 1, 2, and 3) [4].

**Fig. 4:**
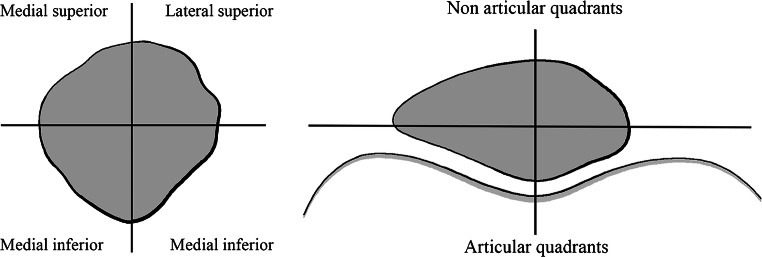
Scheme of the patellar grid. The patella was divided into four articular and four non-articular quadrants to localise the rmBTU on three-dimensional reconstructed SPECT/CT images

For SPECT/CT, each patient received a commercial 700-MBq (18.92 mCi) 99m-Tc-HDP injection (Malinckrodt, Wollerau, Switzerland). SPECT/CT was performed using a hybrid system (Symbia T16, Siemens, Erlangen, Germany), which consists of a pair of low-energy, high-resolution collimators and a dual-head gamma camera with an integrated 16-slice CT scanner (collimation of 16 × 0.75 mm) (Fig. 3). Planar scintigraphic images were taken in the perfusion, in the soft tissue and in the delayed metabolic phase. SPECT/CT was performed with a matrix size of 128 × 128, an angle step of 32, and a time per frame of 25 second, two hours after injection. SPECT/CT images were analysed using a customised software (OrthoExpert©, London, UK). This software has been previously validated in several studies [9, 11, 12]. In order to localise the in vivo bone stress, the patella was divided into four articular and four non-articular quadrants (superomedial, superolateral, inferomedial, inferolateral) (Figs. 3 and 4). This was done according to a previously validated analysis scheme [9]. Absolute BTU values were normalised using the background BTU of the ipsilateral femoral shaft. Relative mean BTU values (rmBTU) were calculated from absolute maximal BTU values on three-dimensional reconstructed CT images in each patellar quadrant. These measurements were done twice by two orthopaedic surgeons with an interval of six weeks. Means of the rmBTU values were used for the following analysis.

As the position of TKA components as well as the patella is known to influence the bone stress at the patella [9, 13], the following parameters were measured on three-dimensional reconstructed CT images and on standardised radiographs: TKA component position, patellar height (modified Insall-Salvati index and Caton-Deschamps index), patellar tilt, patellar thickness, and TT-TG.

The knee function was evaluated with the Knee Society Score (KSS) at three time points: pre-operatively, and at 12 and 24 months after TKA.

Ethical approval was obtained from the Ethics Committee of Northwestern and Central Switzerland (EKNZ 2016-01890). All procedures performed were in accordance with the ethical standards of the institutional and/or national research committee and with the 1964 Declaration of Helsinki and its later amendments or comparable ethical standards. Informed consent was obtained from all individual participants included in the study.

### Statistics

The comparability of the two groups with regard to age, gender, and side was tested using a chi^2^ test. The inter-observer reliability of the rmBTU measurements was evaluated with the single-measures intraclass correlation coefficients (ICC) for every patellar region. The ICC showed excellent correlations between measurements of the two observers (Table 1).

A Mann-Whitney *U* test was performed to investigate significant differences between the two groups with respect to rmBTU of each quadrant, KSS scores, time surgery-SPECT/CT, TKA component position, and patellar measurements. A non-parametric Spearman correlation was performed in each group among all data. A post hoc power analysis showed that with the given *N* (41) and the given allocation ratio between the groups (21/20), an effect size *d* = 0.92 can be shown with a power of 80% (two sided, *p* < 0.05). For 21/20 patients and a two-sided hypothesis, correlations of rho ≥ 0.55/56 or rho ≤ − 0.55/− 0.56 can be found with a power of 80%.

All data were analysed by an independent professional statistician using SPSS™ for Windows 24 (Armonk, NY: IBM Corp, USA) and G*Power 3.1.9 (HHU Düsseldorf). The level of statistical significance was *p* < 0.05.

## Results

A significantly higher in vivo bone stress (rmBTU) was seen in the cruciate-retaining ATTUNE ® Primary Total Knee System (group A) in the lateral non-articular patellar quadrants (Table 1) compared with cruciate-retaining Press-Fit Condylar® Total Knee System (group PFC). This was the only significant difference between the bone stress of both groups (Table 1).

The post-operative KSS scores at one and two years did not differ significantly between groups (Table 1). The TKA alignment differed only for a slightly increased femoral flexion and tibial varus in group A (Table 1). Despite significant higher ratios in group A, the modified Insall-Salvati index and the Caton-Deschamps index were within the accepted normal ranges in both groups (Table 1).

The Spearman correlation showed no correlations between the significantly higher rmBTU of the lateral non-articular patellar quadrants and the position of the TKA and patellar measurements (Tables 2 and 3). The increased flexion of the femoral TKA component in group A did not correlate with rmBTU in any of the eight different patellar quadrants. The increased tibial varus position of group A significantly correlated with increased rmBTU of the articular lateral superior patellar quadrant. The rmBTU was not significantly different between groups A and PFC (Table 1). All patellar measurements did not correlate with bone stress in SPECT/CT (Tables 2 and 3).

**Table 2:** Spearman’s correlations between all collected data of group PFC

Spearman rho-PFC	Rotation femur	Coronal femur	Sagittal femur	Rotation tibia	Coronal tibia	Sagittal tibia	Tilt patella	Patellar thickness	Modified Insall-Salvati index	Caton-Deschamps index	TT-TG patella	KSS total preop	KSS total post12m	KSS total post24m	rmBTU nali	rmBTU ali	rmBTU nals	rmBTU als	rmBTU nami	rmBTU ami	rmBTU nams	rmBTU ams
Rotation femur	1	0	− 0.29	0.25	0.47*	0.45*	− 0.22	− 0.31	− 0.03	− 0.06	0.31	− 0.17	0.2	0.22	− 0.18	− 0.37	− 0.36	− 0.3	− 0.2	− 0.23	− 0.11	− 0.15
Coronal femur	0	1	− 0.01	0.3	− 0.1	0.35	− 0.45	0.5*	− 0.24	− 0.4	− 0.04	− 0.13	0.19	0.07	0.33	0.36	0.36	0.28	0.14	0.11	0.03	0.07
Sagittal femur	− 0.29	− 0.01	1	− 0.4	− 0.13	0.08	0.4	0.17	− 0.05	− 0.02	0.12	− 0.03	− 0.1	0.01	− 0.06	− 0.07	− 0.11	− 0.17	− 0.08	− 0.15	− 0.03	− 0.16
Rotation tibia	0.25	0.3	− 0.4	1	0.06	0	− 0.13	− 0.06	− 0.39	− 0.02	0.22	− 0.32	0.53*	0.07	0.29	0.29	0.22	0.34	0.27	0.38	0.18	0.41
Coronal tibia	0.47*	− 0.1	− 0.13	0.06	1	0.13	0.04	− 0.29	− 0.05	− 0.16	− 0.09	− 0.14	0.37	− 0.36	− 0.01	− 0.18	− 0.04	− 0.11	0.12	− 0.12	0.06	− 0.05
Sagittal tibia	0.45*	0.35	0.08	0	0.13	1	− 0.1	0.19	− 0.03	− 0.23	0.34	− 0.25	− 0.02	0.18	− 0.17	− 0.34	− 0.33	− 0.38	− 0.21	− 0.4	− 0.14	− 0.33
Tilt patella	− 0.22	− 0.45	0.4	− 0.13	0.04	− 0.1	1	− 0.31	0.26	0.43	0.08	− 0.36	− 0.09	− 0.14	− 0.1	− 0.17	− 0.22	− 0.15	0.08	0.08	− 0.05	0
Patellar thickness	− 0.31	0.5*	0.17	− 0.06	− 0.29	0.19	− 0.31	1	0.02	0.03	0.03	0.35	− 0.33	0.04	− 0.02	0.17	0.33	0.26	− 0.19	− 0.27	− 0.15	− 0.19
Modified Insall-Salvati index	− 0.03	− 0.24	− 0.05	− 0.39	− 0.05	− 0.03	0.26	0.02	1	0.48*	0.22	0.42	− 0.22	− 0.02	− 0.24	− 0.28	− 0.19	− 0.13	− 0.15	− 0.07	− 0.23	− 0.13
Caton-Deschamps index	− 0.06	− 0.4	− 0.02	− 0.02	− 0.16	− 0.23	0.43	0.03	0.48*	1	0.11	− 0.06	− 0.1	0	− 0.02	− 0.04	0.08	0.1	− 0.02	0.04	0	0.02
TT-TG patella	0.31	− 0.04	0.12	0.22	− 0.09	0.34	0.08	0.03	0.22	0.11	1	0.04	0.08	0.05	− 0.06	− 0.07	− 0.15	0.04	0.03	0.1	0.08	0.2
KSS total preop	− 0.17	− 0.13	− 0.03	− 0.32	− 0.14	− 0.25	− 0.36	0.35	0.42	− 0.06	0.04	1	0.05	0.11	− 0.1	0.02	0.07	0.11	− 0.22	− 0.05	− 0.14	0.01
KSS total post12m	0.2	0.19	− 0.1	0.53*	0.37	− 0.02	− 0.09	− 0.33	− 0.22	− 0.1	0.08	0.05	1	0.12	0.37	0.29	0.27	0.17	0.3	0.29	0.23	0.34
KSS total post24m	0.22	0.07	0.01	0.07	− 0.36	0.18	− 0.14	0.04	− 0.02	0	0.05	0.11	0.12	1	− 0.24	− 0.27	− 0.26	− 0.32	− 0.41	− 0.28	− 0.12	− 0.13
rmBTU nali	− 0.18	0.33	− 0.06	0.29	− 0.01	− 0.17	− 0.1	− 0.02	− 0.24	− 0.02	− 0.06	− 0.1	0.37	− 0.24	1	0.91***	0.85***	0.74***	0.87***	0.81***	0.85***	0.8***
rmBTU ali	− 0.37	0.36	− 0.07	0.29	− 0.18	− 0.34	− 0.17	0.17	− 0.28	− 0.04	− 0.07	0.02	0.29	− 0.27	0.91***	1	0.93***	0.9***	0.8***	0.83***	0.75***	0.82***
rmBTU nals	− .36	0.36	− 0.11	0.22	− 0.04	− 0.33	− 0.22	0.33	− 0.19	0.08	− 0.15	0.07	0.27	− 0.26	0.85***	0.93***	1	0.9***	0.71***	0.69***	0.7***	0.71***
rmBTU als	− 0.3	0.28	− 0.17	0.34	− 0.11	− 0.38	− 0.15	0.26	− 0.13	0.1	0.04	0.11	0.17	− 0.32	0.74***	0.9***	0.9***	1	0.65***	0.77***	0.61**	0.78***
rmBTU nami	− 0.2	0.14	− 0.08	0.27	0.12	− 0.21	0.08	− 0.19	− 0.15	− 0.02	0.03	− 0.22	0.3	− 0.41	0.87***	0.8***	0.71***	0.65***	1	0.9***	0.89***	0.87***
rmBTU ami	− 0.23	0.11	− 0.15	0.38	− 0.12	− 0.4	0.08	− 0.27	− 0.07	0.04	0.1	− 0.05	0.29	− 0.28	0.81***	0.83***	0.69***	0.77***	0.9***	1	0.8***	0.95***
rmBTU nams	− 0.11	0.03	− 0.03	0.18	0.06	− 0.14	− 0.05	− 0.15	− 0.23	0	0.08	− 0.14	0.23	− 0.12	0.85***	0.75***	0.7***	0.61**	0.89***	0.8***	1	0.88***
rmBTU ams	− 0.15	0.07	− 0.16	0.41	− 0.05	− 0.33	0	− 0.19	− 0.13	0.02	0.2	0.01	0.34	− 0.13	0.8***	0.82***	0.71***	0.78***	0.87***	0.95***	0.88***	1

*p < 0.05

**p < 0.01

***p < 0.001

**Table 3:** Spearman’s correlations between all collected data for group A

Spearman rho-Attune	Rotation femur	Coronal femur	Sagittal femur	Rotation tibia	Coronal tibia	Sagittal tibia	Tilt patella	Patellar thickness	Modified Insall-Salvati index	Caton-Deschamps index	TT-TG patella	KSS total preop	KSS total post12m	KSS total post24m	rmBTU nali	rmBTU ali	rmBTU nals	rmBTU als	rmBTU nami	rmBTU ami	rmBTU nams	rmBTU ams
Rotation femur	1	− 0.32	− 0.09	− 0.45*	0.4	0.09	0.13	0.54	0.17	0.23	0.21	− 0.32	− 0.06	− 0.07	0.1	0.01	0.05	− 0.02	0.17	0.19	0.08	0.28
Coronal femur	− 0.32	1	− 0.07	0.1	− 0.33	− 0.19	0.23	0.32	0.12	0.26	− 0.03	0.31	− 0.16	− 0.28	0.48*	0.37	0.31	0.31	0.07	0.02	0.09	− 0.07
Sagittal femur	− 0.09	− 0.07	1	− 0.26	0.11	0.05	− 0.26	− 0.13	0.11	0.05	0.09	− 0.08	0.27	0.43	0.02	− 0.03	0.04	− 0.03	0.25	0.24	− 0.19	0.06
Rotation tibia	− 0.45*	0.1	− 0.26	1	− 0.34	0.04	0.02	− 0.15	− 0.04	− 0.39	− 0.1	− 0.1	− 0.13	− 0.37	− 0.09	− 0.1	− 0.03	− 0.03	− 0.1	− 0.12	0.13	0.02
Coronal tibia	0.4	− 0.33	0.11	− 0.34	1	− 0.19	− 0.28	0.04	0.13	0.29	0.43	− 0.04	0.38	0.56*	− 0.26	− 0.39	− 0.33	− 0.51*	0.03	− 0.22	− 0.16	− 0.35
Sagittal tibia	0.09	− 0.19	0.05	0.04	− 0.19	1	0.53	0.15	− 0.2	− 0.14	0.07	− 0.25	− 0.16	− 0.09	− 0.26	− 0.24	− 0.16	− 0.18	− 0.05	− 0.02	− 0.09	− 0.02
Tilt patella	0.13	0.23	− 0.26	0.02	− 0.28	0.53	1	0.49	− 0.37	− 0.07	0.2	− 0.23	− 0.44	− 0.86**	− 0.07	− 0.16	− 0.22	− 0.42	− 0.2	0.01	− 0.11	− 0.21
Patellar thickness	0.54	0.32	− 0.13	− 0.15	0.04	0.15	0.49	1	− 0.11	− 0.04	0.63*	− 0.31	− 0.07	− 0.42	0.26	0.14	0.15	− 0.24	0.06	0.08	− 0.08	− 0.15
Modified Insall-Salvati index	0.17	0.12	0.11	− 0.04	0.13	− 0.2	− 0.37	− 0.11	1	0.6**	0.12	0.37	0.15	0.22	0	− 0.02	− 0.07	0.1	− 0.09	− 0.06	− 0.18	0.04
Caton-Deschamps index	0.23	0.26	0.05	− 0.39	0.29	− 0.14	− 0.07	− 0.04	0.6**	1	0.03	0.62**	0.26	0.18	0.07	0.01	− 0.05	0.02	0.01	− 0.1	− 0.19	− 0.13
TT-TG patella	0.21	− 0.03	0.09	− 0.1	0.43	0.07	0.2	0.63*	0.12	0.03	1	0	0.19	0.22	− 0.24	− 0.34	− 0.18	− 0.56*	− 0.1	− 0.26	− 0.11	− 0.33
KSS total preop	− 0.32	0.31	− 0.08	− 0.1	− 0.04	− 0.25	− 0.23	− 0.31	0.37	0.62**	0	1	0.27	0.01	0.23	0.18	0.22	0.13	0.01	− 0.15	0.03	− 0.21
KSS total post12m	− 0.06	− 0.16	0.27	− 0.13	0.38	− 0.16	− 0.44	− 0.07	0.15	0.26	0.19	0.27	1	0.68**	0.1	0.17	0.07	0.03	0.2	0.21	− 0.23	− 0.15
KSS total post24m	− 0.07	− 0.28	0.43	− 0.37	0.56*	− 0.09	− 0.86**	− 0.42	0.22	0.18	0.22	0.01	0.68**	1	− 0.21	− 0.07	− 0.29	− 0.19	− 0.07	− 0.1	− 0.54*	− 0.42
rmBTU nali	0.1	0.48*	0.02	− 0.09	− 0.26	− 0.26	− 0.07	0.26	0	0.07	− 0.24	0.23	0.1	− 0.21	1	0.76***	0.9***	0.65**	0.65**	0.48*	0.47*	0.27
rmBTU ali	0.01	0.37	− 0.03	− 0.1	− 0.39	− 0.24	− 0.16	0.14	− 0.02	0.01	− 0.34	0.18	0.17	− 0.07	0.76***	1	0.65**	0.77***	0.24	0.61**	0.05	0.42
rmBTU nals	0.05	0.31	0.04	− 0.03	− 0.33	− 0.16	− 0.22	0.15	− 0.07	− 0.05	− 0.18	0.22	0.07	− 0.29	0.9***	0.65**	1	0.7***	0.72***	0.52*	0.67***	0.47*
rmBTU als	− 0.02	0.31	− 0.03	− 0.03	− 0.51*	− 0.18	− 0.42	− 0.24	0.1	0.02	− 0.56*	0.13	0.03	− 0.19	0.65**	0.77***	0.7***	1	0.33	0.56**	0.37	0.75***
rmBTU nami	0.17	0.07	0.25	− 0.1	0.03	− 0.05	− 0.2	0.06	− 0.09	0.01	− 0.1	0.01	0.2	− 0.07	0.65**	0.24	0.72***	0.33	1	0.6**	0.73***	0.34
rmBTU ami	0.19	0.02	0.24	− 0.12	− 0.22	− 0.02	0.01	0.08	− 0.06	− 0.1	− 0.26	− 0.15	0.21	− 0.1	0.48*	0.61**	0.52*	0.56**	0.6**	1	0.32	0.68***
rmBTU nams	0.08	0.09	− 0.19	0.13	− 0.16	− 0.09	− 0.11	− 0.08	− 0.18	− 0.19	− 0.11	0.03	− 0.23	− 0.54*	0.47*	0.05	0.67***	0.37	0.73***	0.32	1	0.48*
rmBTU ams	0.28	− 0.07	0.06	0.02	− 0.35	− 0.02	− 0.21	− 0.15	0.04	− 0.13	− 0.33	− 0.21	− 0.15	− 0.42	0.27	0.42	0.47*	0.75***	0.34	0.68***	0.48*	1

*p < 0.05

**p < 0.01

***p < 0.001

## Discussion

The most important finding of this study is that, despite technical innovations with regard to the trochlea design, the in vivo bone stress on the articular part of the patella did not significantly change between the cruciate-retaining Press-Fit Condylar® Total Knee System (group PFC) and the cruciate-retaining ATTUNE ® Primary Total Knee System (group A). Significant differences were seen in terms of rmBTU at the non-articular part of the patella with an increased in vivo bone stress in the lateral quadrants. A possible explanation could lie in the design of the femoral trochlea. Besides a thinner femoral trochlea, different trochlear groove angles at every degree of knee flexion are presented as an important benefit of the Attune TKA system. While in full-knee extension the Attune has a flatter trochlea (bigger trochlear sulcus angle) than the PFC (Attune: 157.4°, PFC: 154.5°), it becomes deeper at 15° (Attune: 147.3°, PFC: 152.0°) and at 30° (Attune: 146.7°, PFC: 149.7°) of flexion to return flatter at 45° (Attune: 146.2°, PFC: 140.0°) [6]. It could be speculated that the less patellar constraint of the Attune in full extension could lead to an increased activation of the quadriceps muscle in order to provide axial stability. Furthermore, the increased constraint level of the trochlea at 15° and 30° of flexion did not lead to increased bone stress at the articular part of the patella. On the other hand, the deeper femoral trochlea in the first 30° of flexion for the Attune seems to improve patellar tracking, reducing the need for a lateral release at TKA when compared with the PFC [6]. However, it has to be taken into consideration that even if the Attune has a more anatomical trochlear groove, its 146.2° sulcus angle at 45° flexion exceed for 2 degrees the criteria for trochlear dysplasia [6, 14]. In fact, this could lead to patellar instability/maltracking and has to be considered during TKA [6]. Furthermore, the roles of the medial retinaculum and capsule have also to be taken into account while evaluating the patellar tracking and soft-tissue balance. It has been shown how a temporary closed medial arthrotomy with two provisional stitches in the superior and inferior borders of the patella increases significantly the load on the medial compartment compared with a patella with open arthrotomy [15].

The position of the prosthetic components is another important aspect that has to be mentioned. A suboptimal TKA position is a well-known source of TKA failure [9, 12, 13, 16, 17]. Malpositioned prosthetic components, by altering the physiological biomechanics of the knee and of the lever arm of the extensor muscles, can lead to increased patellar stress. Significant correlations between increased patellar rmBTU and TKA malposition have already been reported [7–9, 18, 19]. In the present study, the three-dimensional position of the prosthetic components as well as the position of the patella have been considered and analysed for significant influences on the patellar bone stress. It has been shown that, on the two quadrants where the two groups presented different rmBTU values, the TKA alignment and the position of the patella had no significant influence. Clearly, this strengthen the importance of the findings, as a possible bias by the TKA position is highly unlikely. It can be stated that the design of the femoral trochlea remains an open issue in knee arthroplasty and that more studies are needed to understand how to improve it.

The second finding of the study was that the both implants achieved good and similar 12- and 24-month post-operative KSS. This is in accordance with the current studies with the largest cohort of patients; Molloy, Martin, and Ranawatt analysed respectively 2116, 1983, and 200 patients who undergone these two types of TKAs and failed to find any significant difference at functional scores [20–23]. Only a significant reduction of patellar crepitus and length of stay in hospital were seen in patients who underwent Attune TKA [20, 22]. Discordant with these results are some minor studies with limited cohort of patients, which showed a little superiority of Attune TKA at functional scores [24, 25].

The major strength of the present study is that, for the first time, a detailed analysis of patellar bone stress of two different TKA systems was performed using SPECT/CT. The main limitation is the small sample size, but this is in line with the sample size calculation done. In addition, it is outweighed by the fact that most confounding factors such as TKA position were considered. Another important limitation is the focus of the study on bone stress without taking into account more dynamic variables such as the tension of the peripatellar soft-tissue structures. Finally, the results are only valid for these two types of TKA systems investigated and cannot be generalised to any other TKA system.

In conclusion, the latest-generation CR-TKA system that has been analysed did not show any significant improvement in terms of in vivo patellar bone stress or knee function compared with its predecessor. No study recommended the use of one of the studied CR-TKA implants for a specific type of knee osteoarthritis.

The increased bone stress seen on SPECT/CT images at the non-articular lateral patellar quadrants of the Attune could be caused from the higher stabilising quadriceps forces needed to compensate the less-constrained trochlea in the first degrees of flexion. The challenge to develop a more patella-friendly femoral trochlea remains open.
